# Supplementary figures and images for: Identification and characterization of novel sesquiterpene synthases TPS9 and TPS12 from Aquilaria sinensis
Source: PeerJ. 2023 Aug 30;11:e15818. doi: 10.7717/peerj.15818 (PMC10474832; doi:10.7717/peerj.15818)

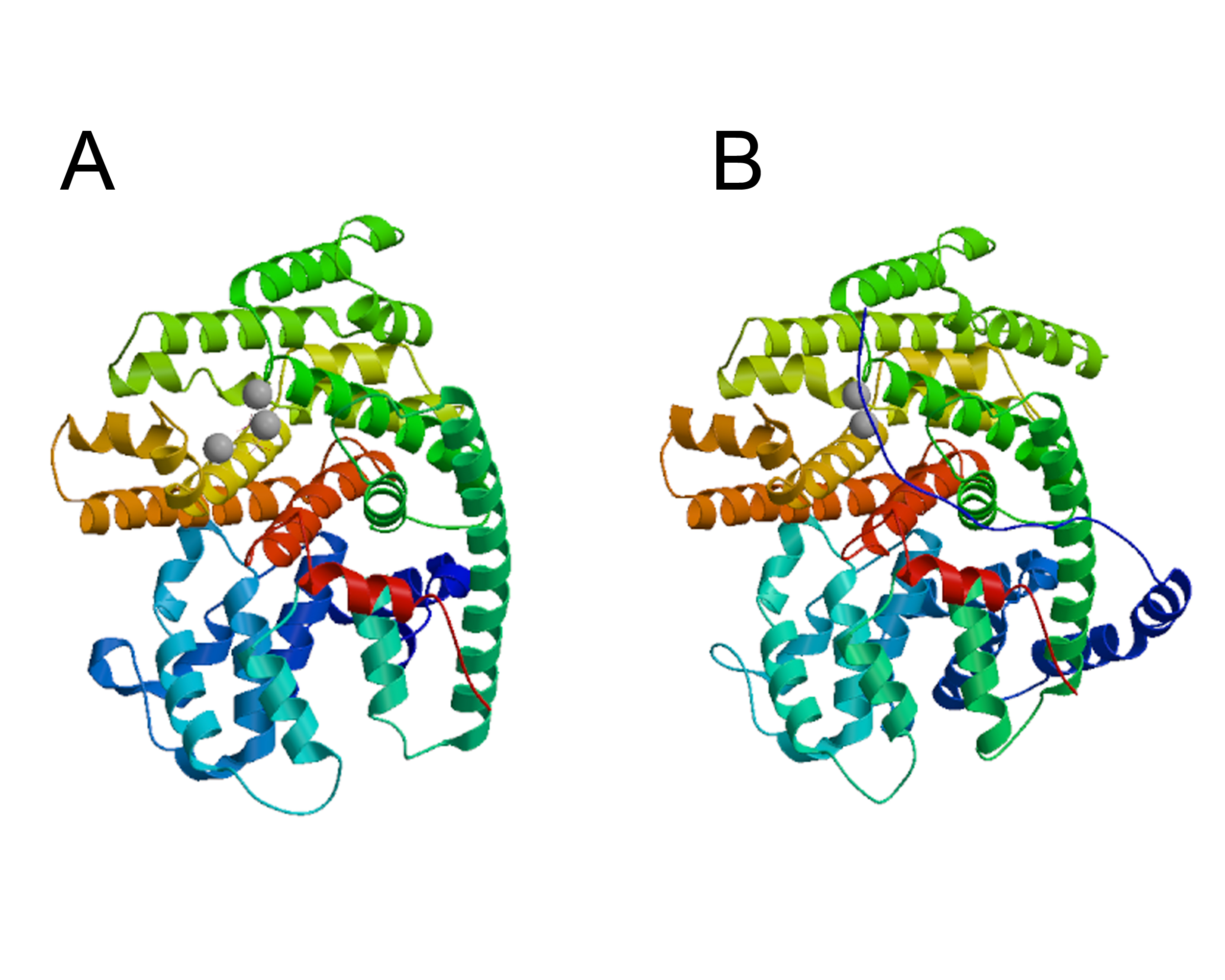

Supplement: Supplemental Information 1 — (A) TPS9. (B) TPS12 [file peerj-11-15818-s001.jpg]

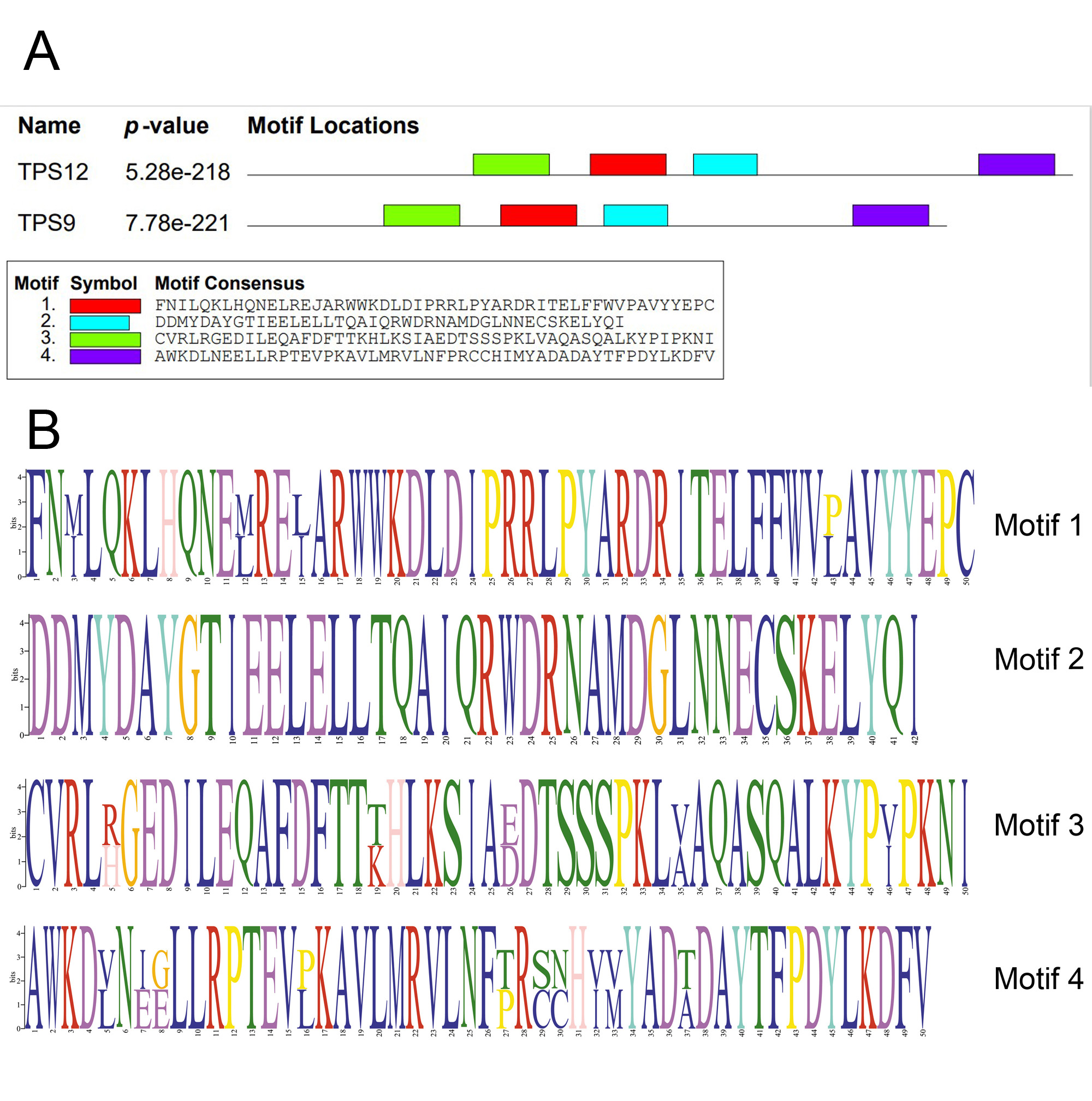

Supplement: Supplemental Information 2 — Conserved motifs identified with the MEME search tool. (A) Motifs are represented by boxes. Different colors boxes represent motif 1–4, respectively and the size of box indicates the length of motifs. (B) The structure diagram of motif 1–4. [file peerj-11-15818-s002.jpg]

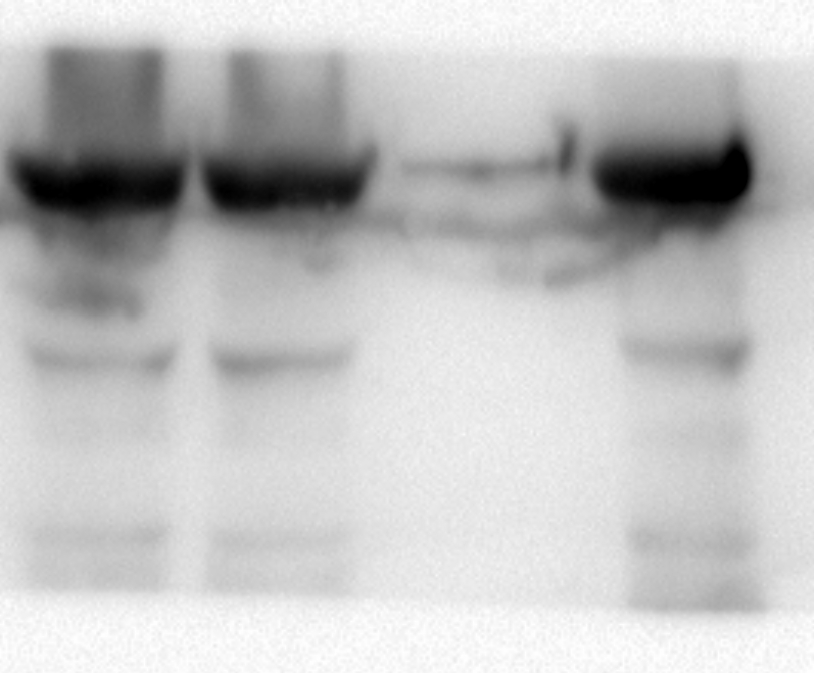

Supplement: Supplemental Information 5 [file peerj-11-15818-s005.zip › full-length uncropped blots of Fig3/TPS12-28A.jpg]

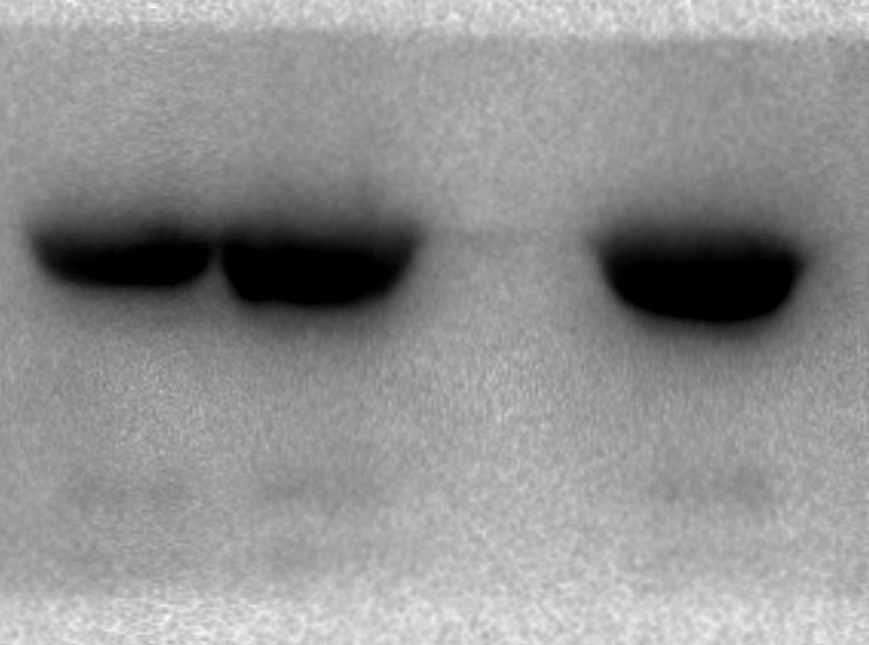

Supplement: Supplemental Information 5 [file peerj-11-15818-s005.zip › full-length uncropped blots of Fig3/TPS9-21A.jpg]

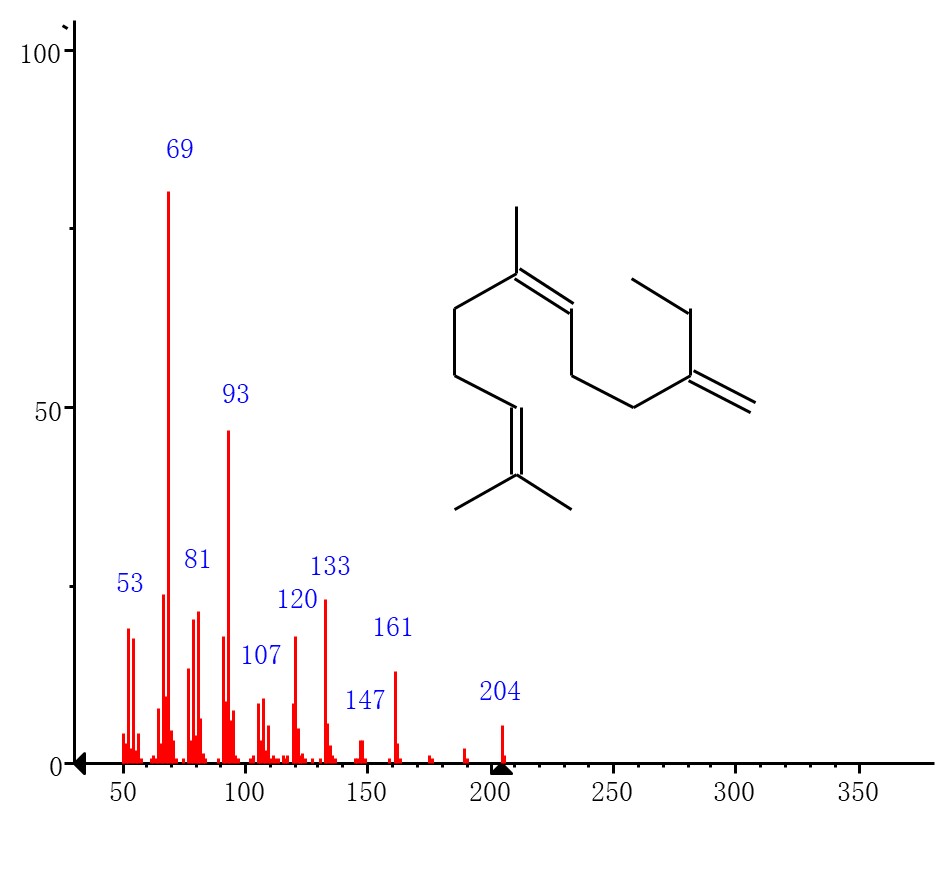

Supplement: Supplemental Information 7 [file peerj-11-15818-s007.zip › GC/TPS12-28A FPP/B1.jpg]

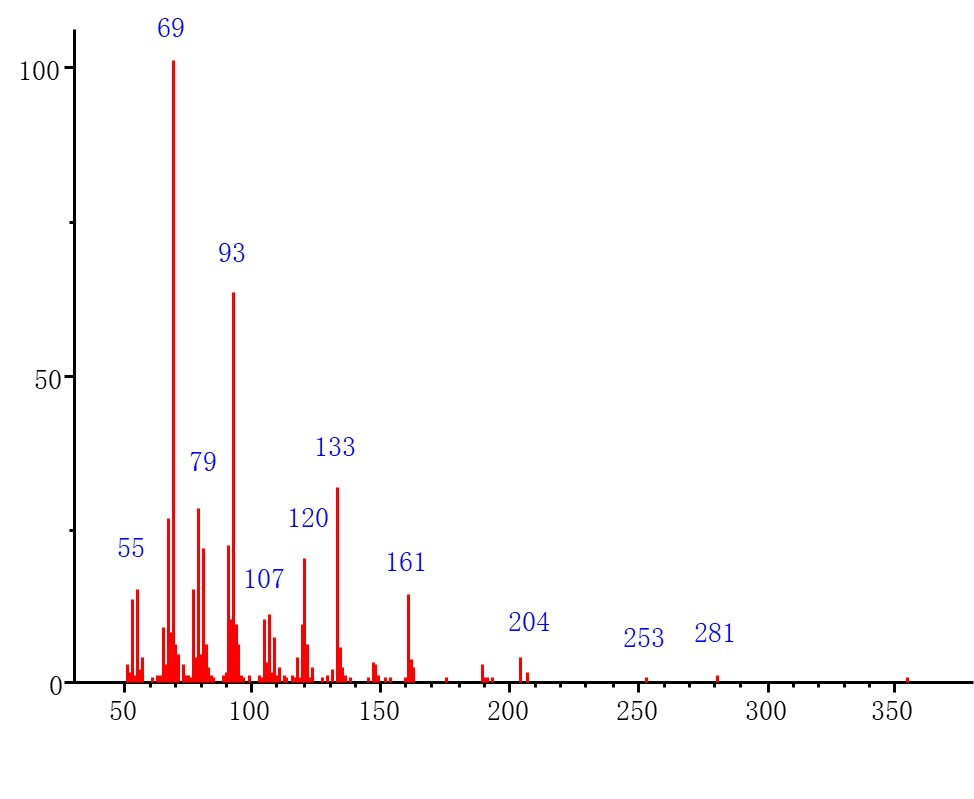

Supplement: Supplemental Information 7 [file peerj-11-15818-s007.zip › GC/TPS12-28A FPP/B2.jpg]

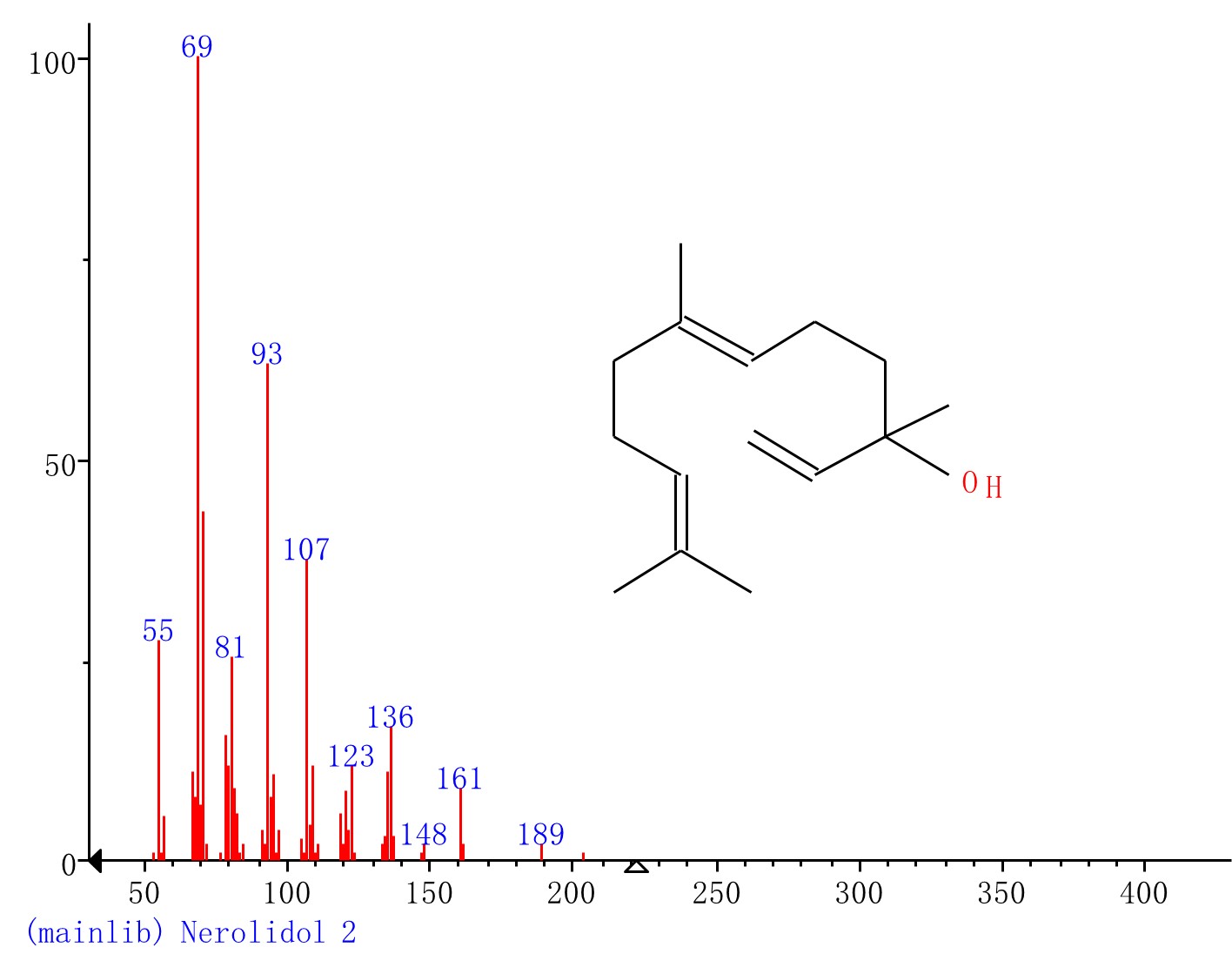

Supplement: Supplemental Information 7 [file peerj-11-15818-s007.zip › GC/TPS12-28A FPP/B3.jpg]

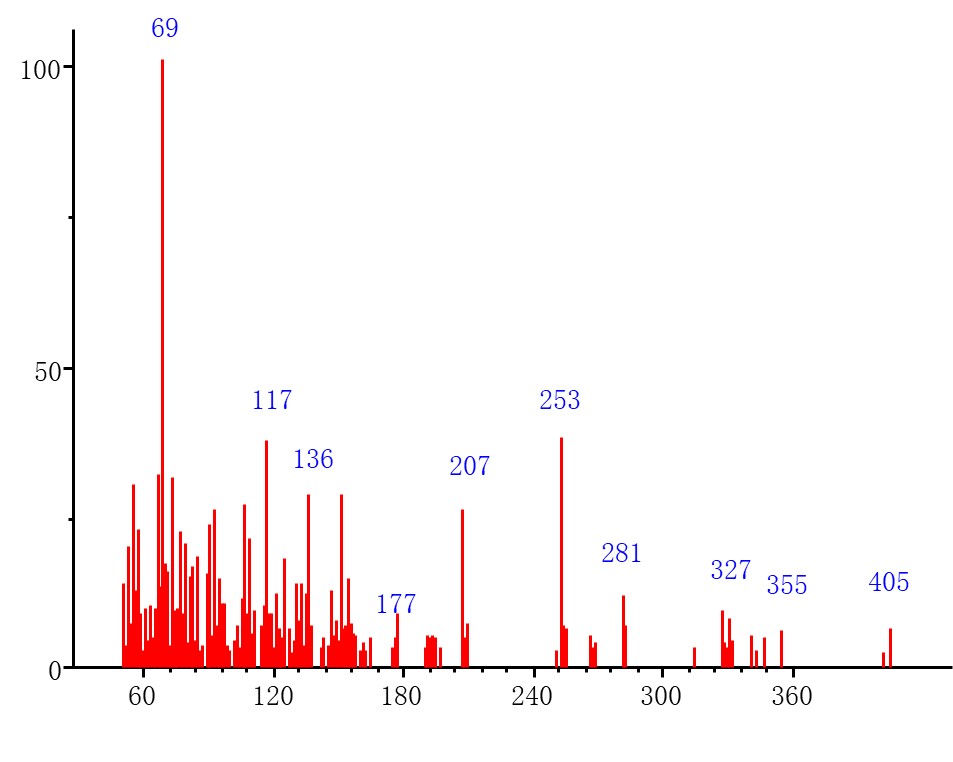

Supplement: Supplemental Information 7 [file peerj-11-15818-s007.zip › GC/TPS12-28A FPP/B4.jpg]

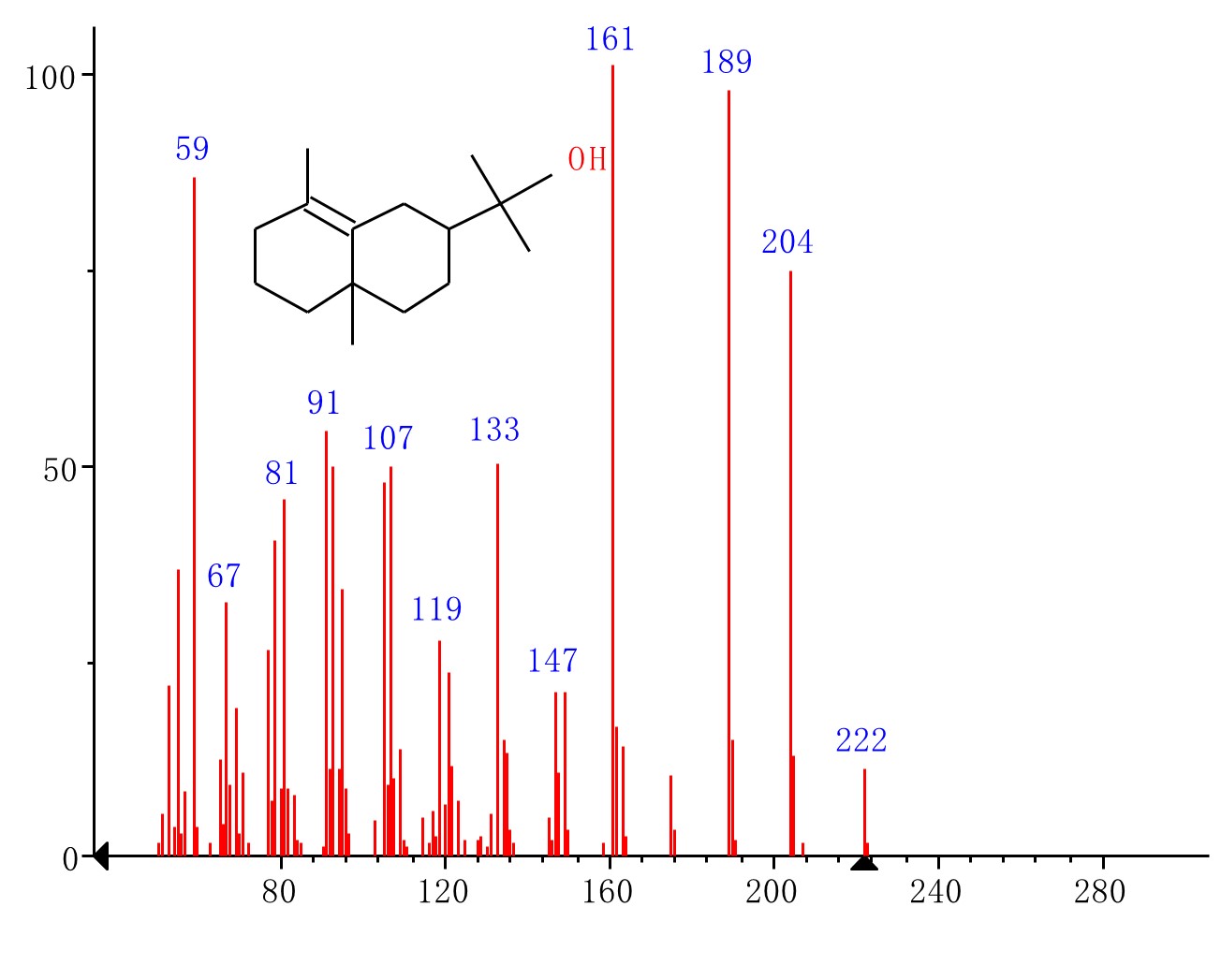

Supplement: Supplemental Information 7 [file peerj-11-15818-s007.zip › GC/TPS12-28A FPP/B5.jpg]

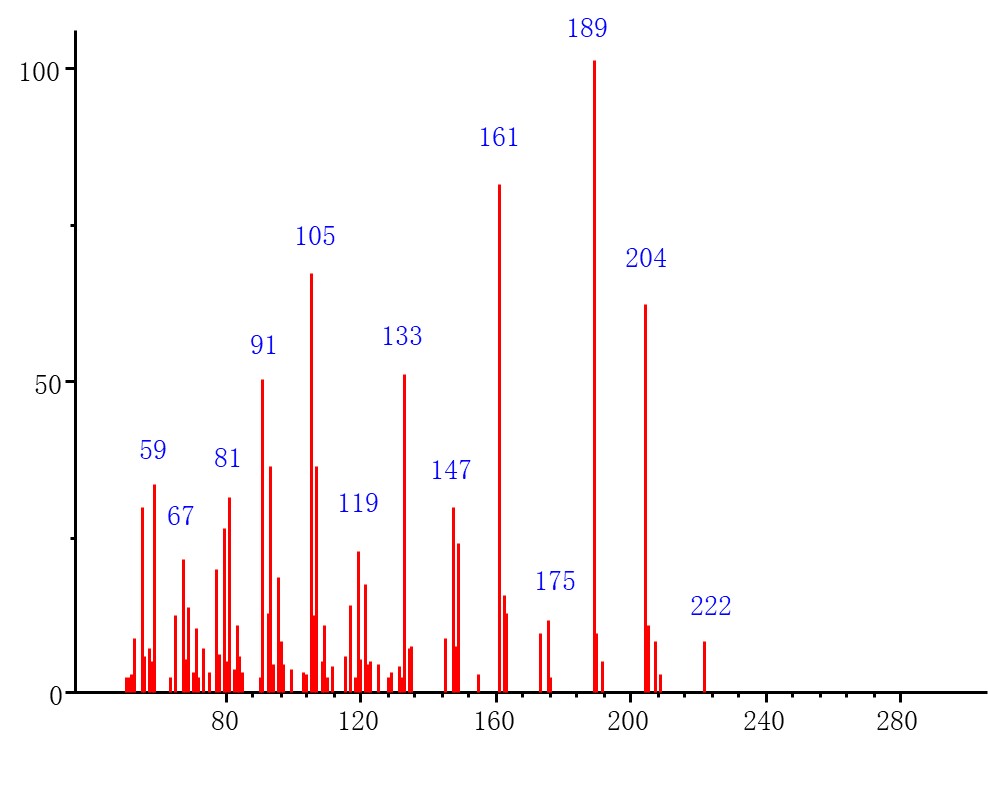

Supplement: Supplemental Information 7 [file peerj-11-15818-s007.zip › GC/TPS12-28A FPP/B6.jpg]

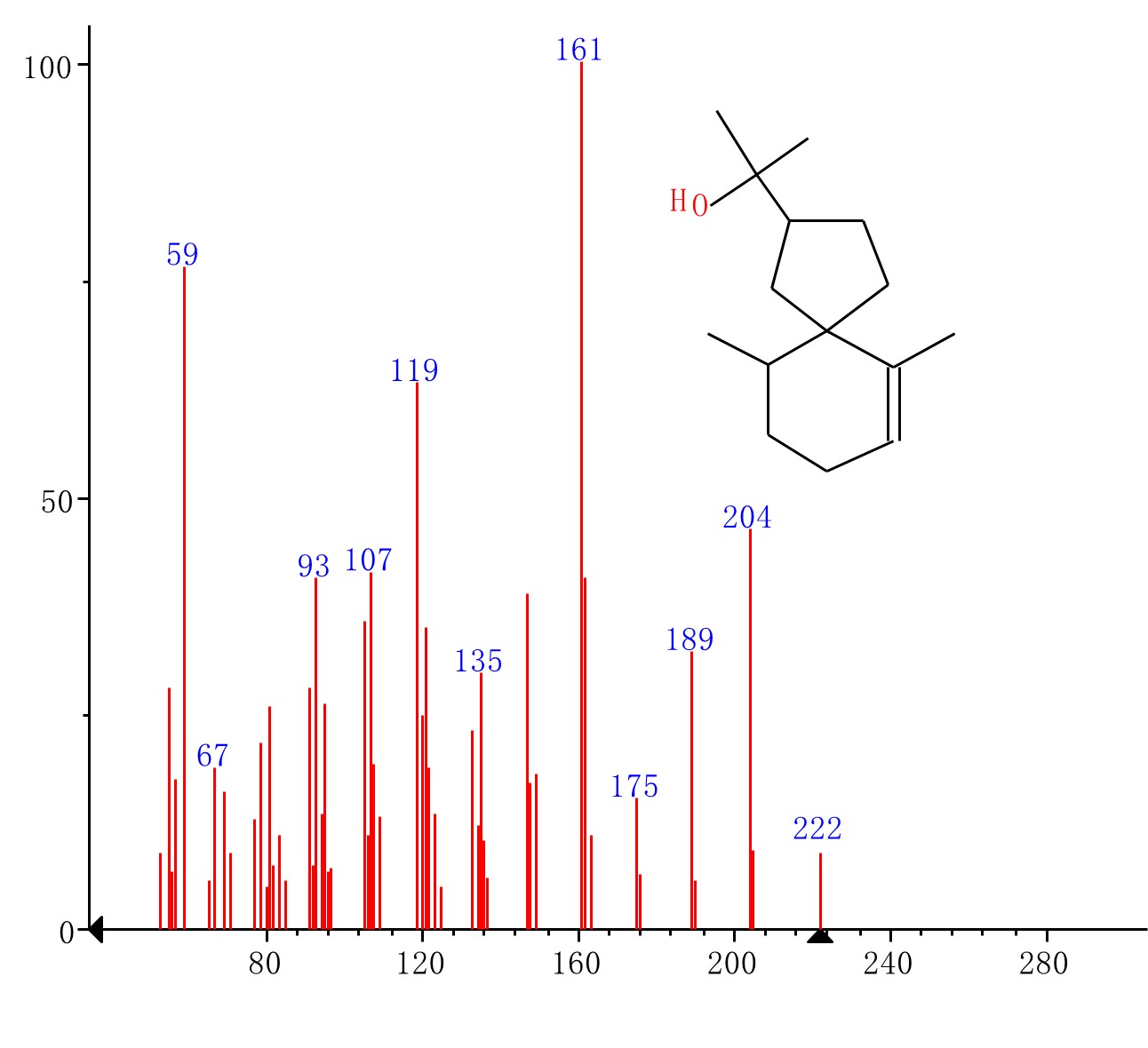

Supplement: Supplemental Information 7 [file peerj-11-15818-s007.zip › GC/TPS12-28A FPP/B7.jpg]

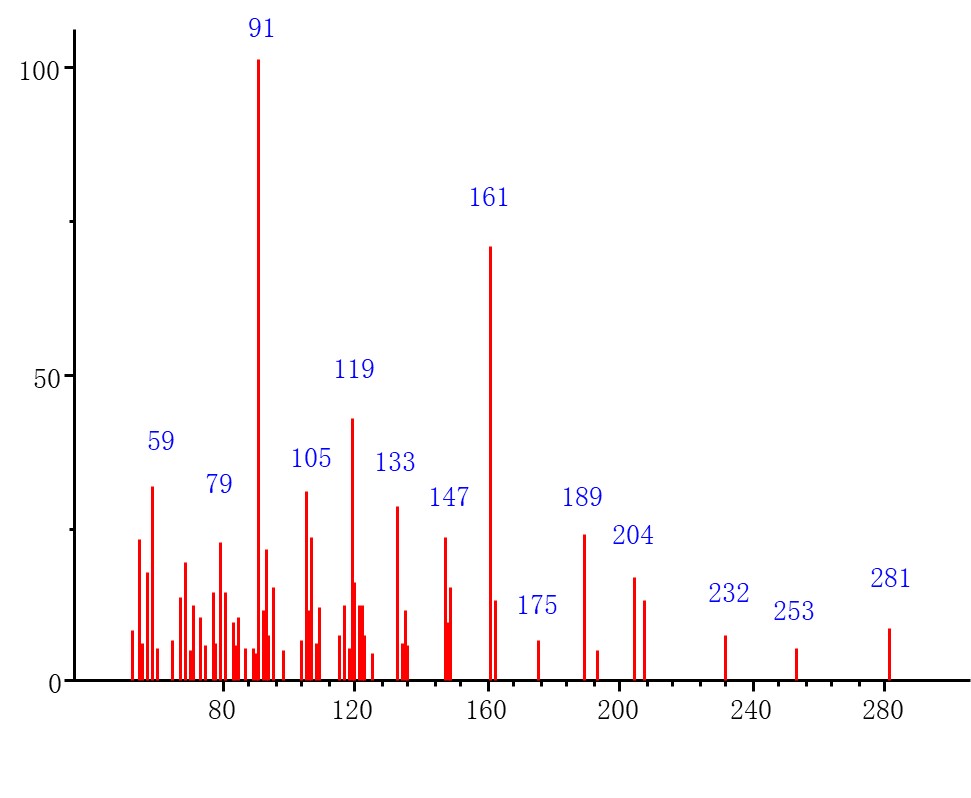

Supplement: Supplemental Information 7 [file peerj-11-15818-s007.zip › GC/TPS12-28A FPP/B8.jpg]

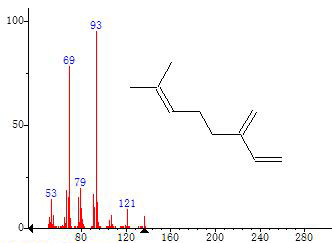

Supplement: Supplemental Information 7 [file peerj-11-15818-s007.zip › GC/TPS12-28a GPP/B1.jpg]

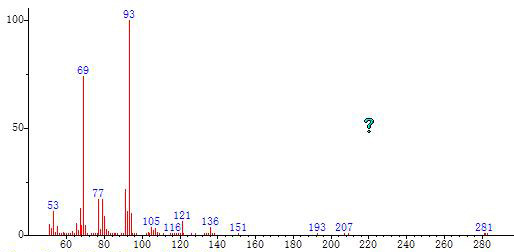

Supplement: Supplemental Information 7 [file peerj-11-15818-s007.zip › GC/TPS12-28a GPP/B2.jpg]

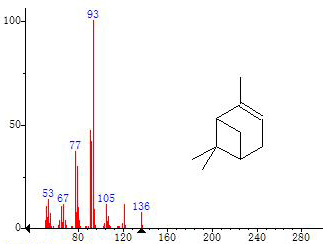

Supplement: Supplemental Information 7 [file peerj-11-15818-s007.zip › GC/TPS12-28a GPP/B3.jpg]

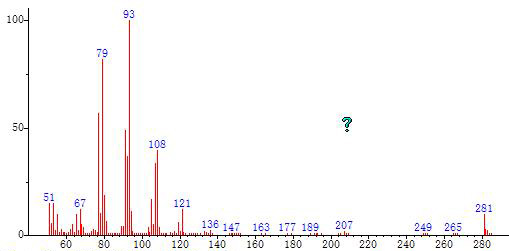

Supplement: Supplemental Information 7 [file peerj-11-15818-s007.zip › GC/TPS12-28a GPP/B4.jpg]

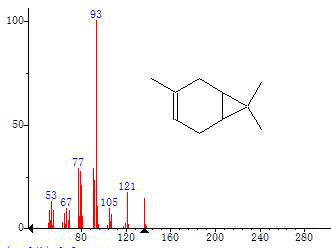

Supplement: Supplemental Information 7 [file peerj-11-15818-s007.zip › GC/TPS12-28a GPP/B5.jpg]

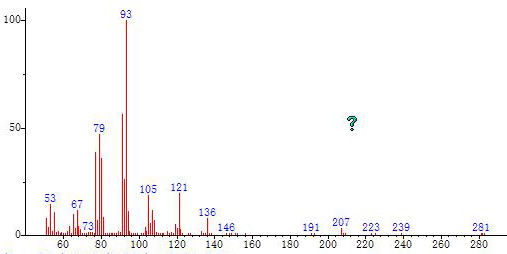

Supplement: Supplemental Information 7 [file peerj-11-15818-s007.zip › GC/TPS12-28a GPP/B6.jpg]

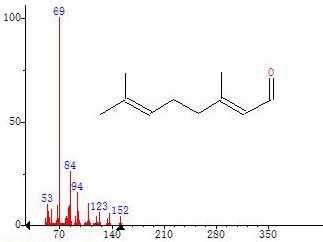

Supplement: Supplemental Information 7 [file peerj-11-15818-s007.zip › GC/TPS12-28a GPP/B7.jpg]

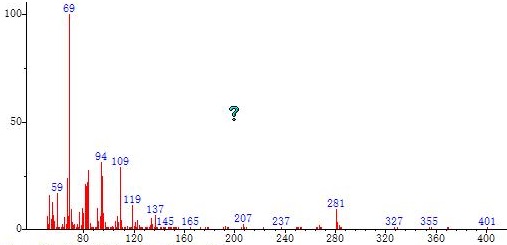

Supplement: Supplemental Information 7 [file peerj-11-15818-s007.zip › GC/TPS12-28a GPP/B8.jpg]

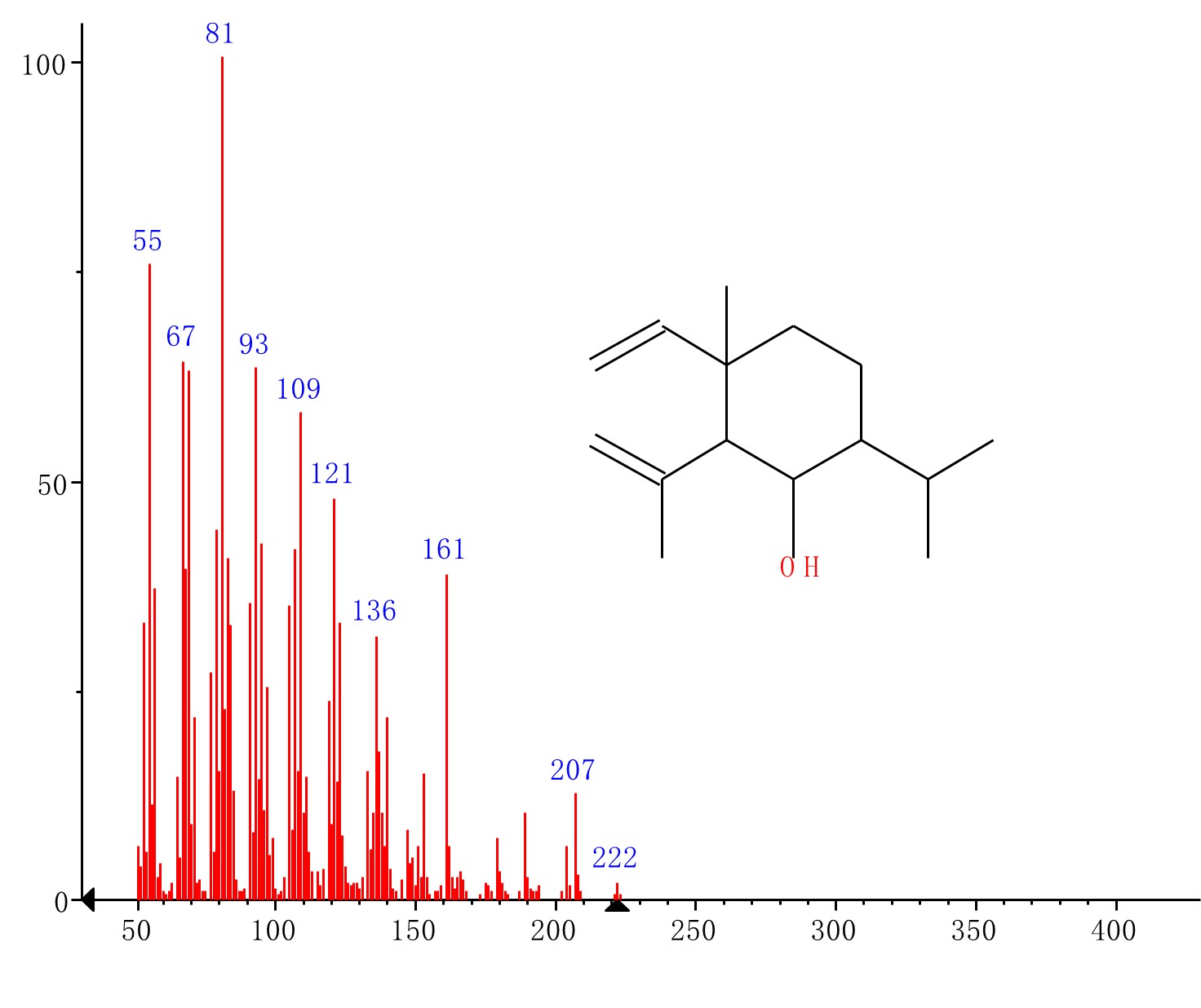

Supplement: Supplemental Information 7 [file peerj-11-15818-s007.zip › GC/TPS9-21A FPP/A1.jpg]

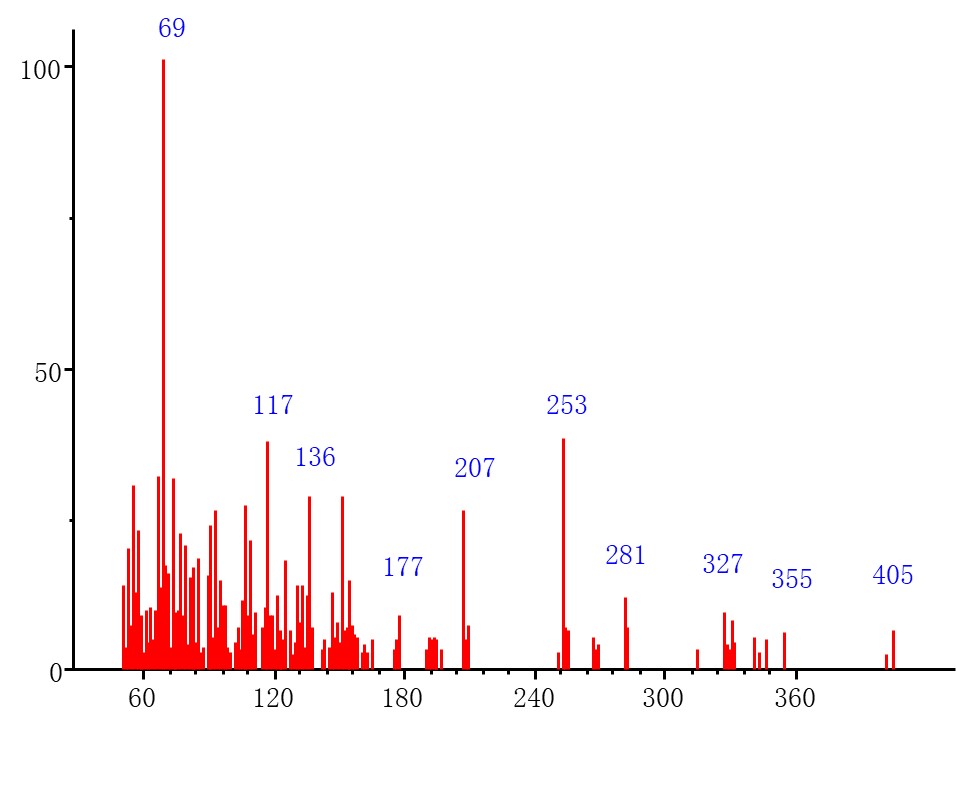

Supplement: Supplemental Information 7 [file peerj-11-15818-s007.zip › GC/TPS9-21A FPP/A2.jpg]

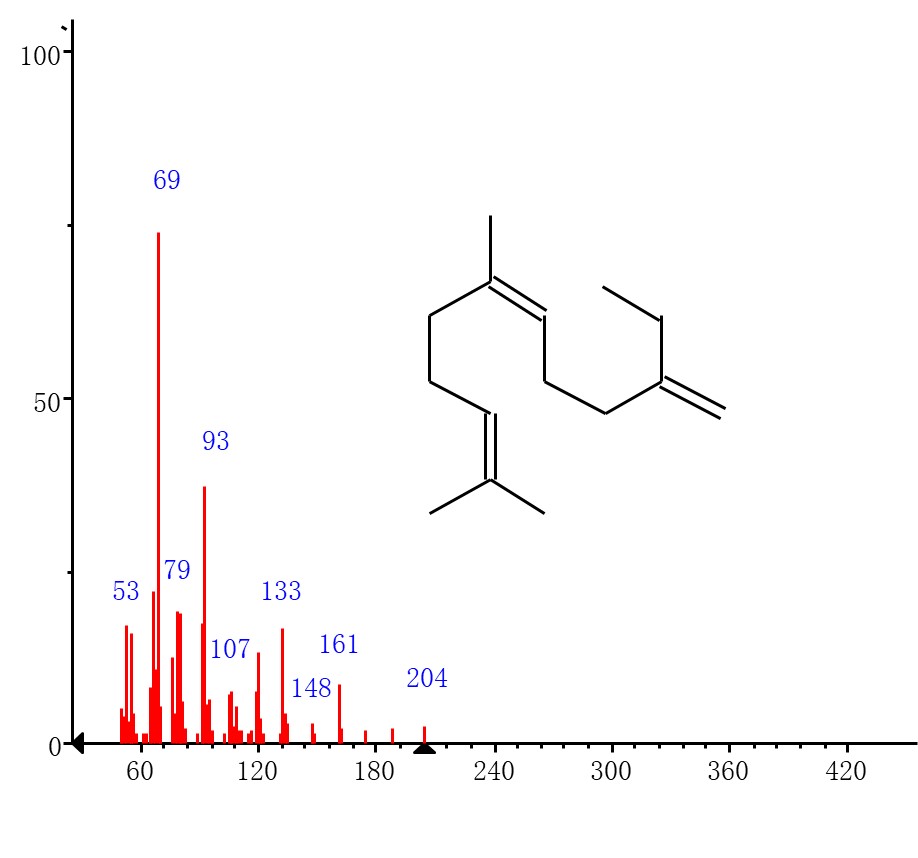

Supplement: Supplemental Information 7 [file peerj-11-15818-s007.zip › GC/TPS9-21A FPP/A3.jpg]

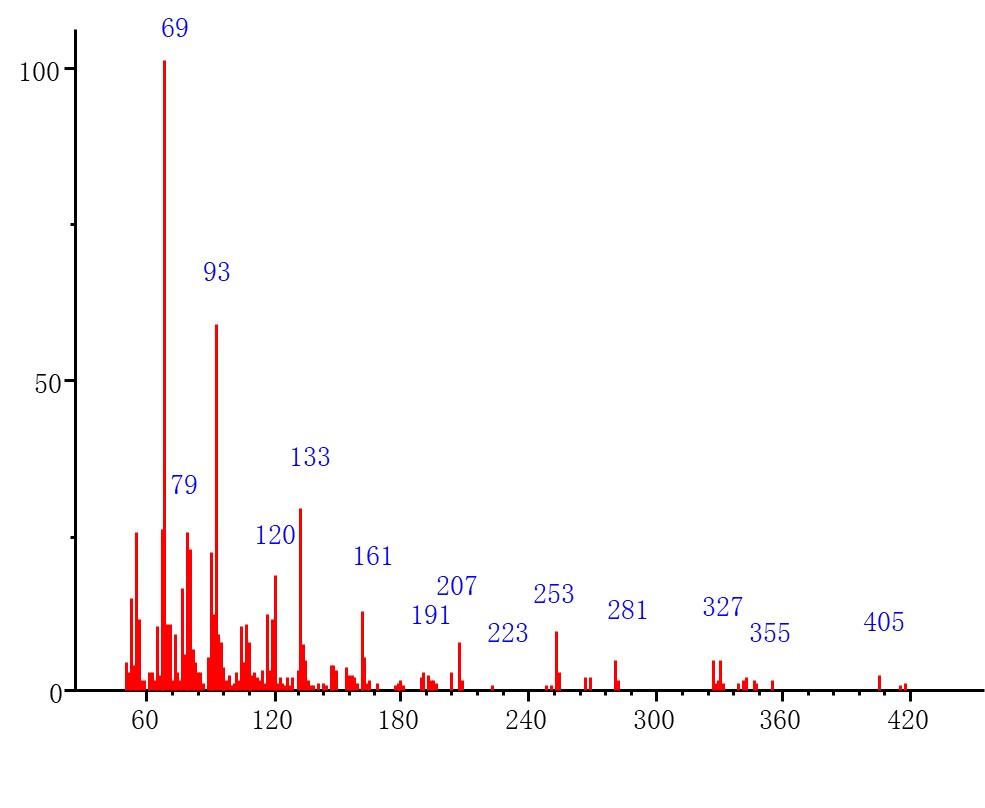

Supplement: Supplemental Information 7 [file peerj-11-15818-s007.zip › GC/TPS9-21A FPP/A4.jpg]

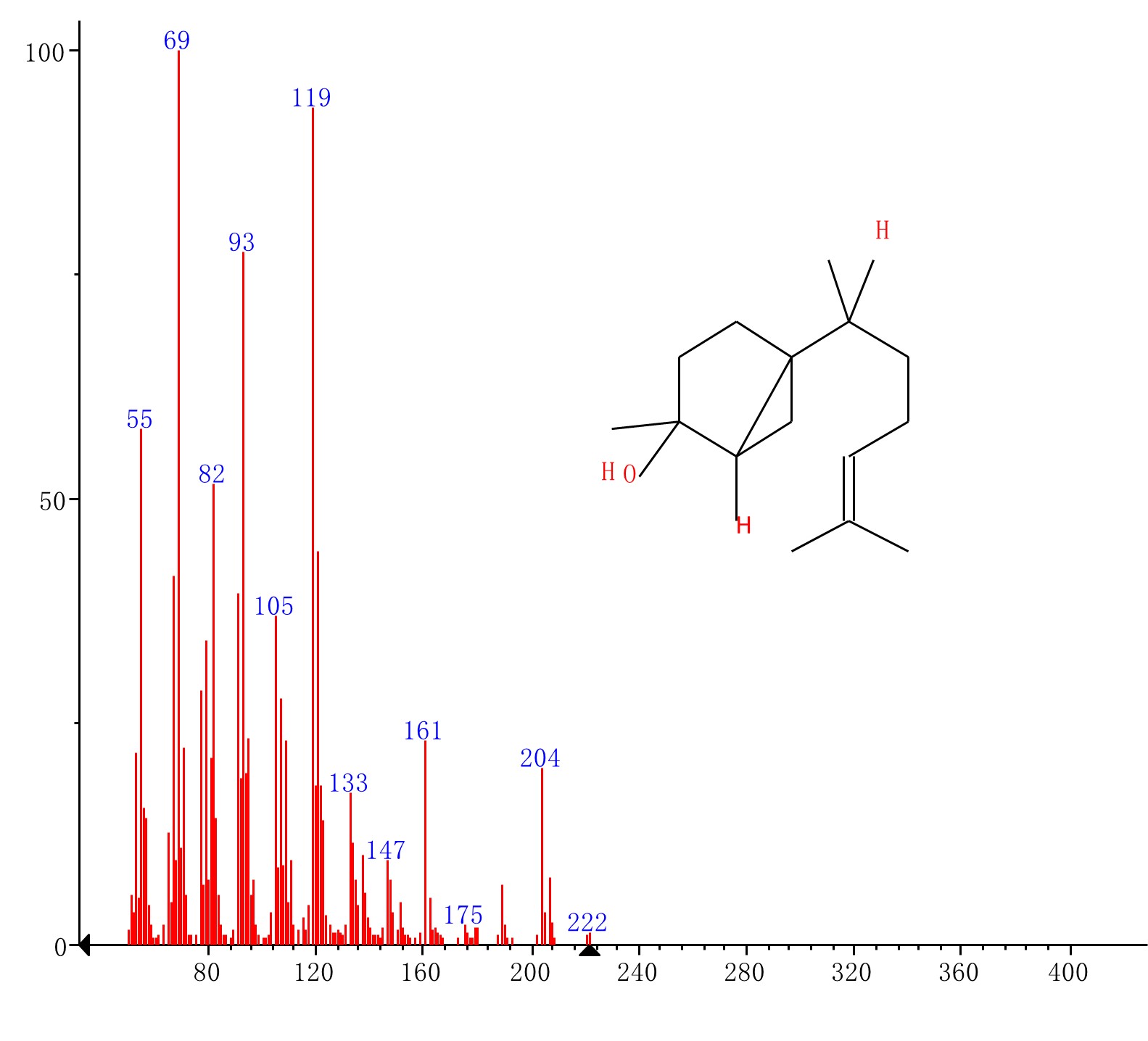

Supplement: Supplemental Information 7 [file peerj-11-15818-s007.zip › GC/TPS9-21A FPP/A5.jpg]

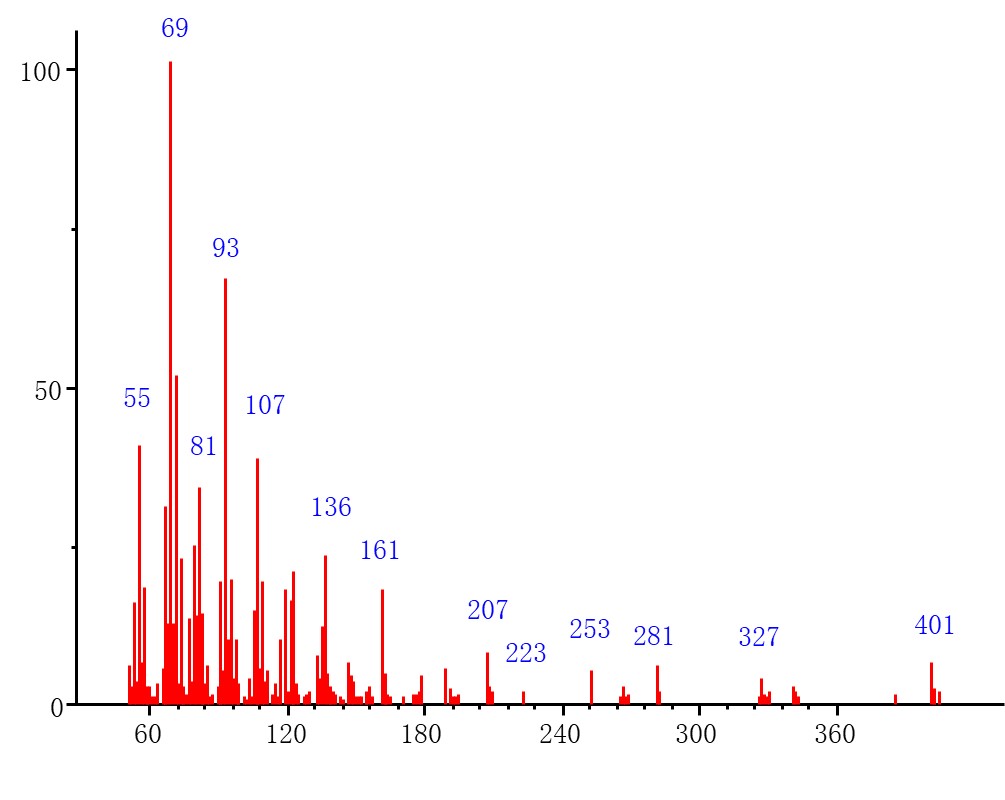

Supplement: Supplemental Information 7 [file peerj-11-15818-s007.zip › GC/TPS9-21A FPP/A6.jpg]

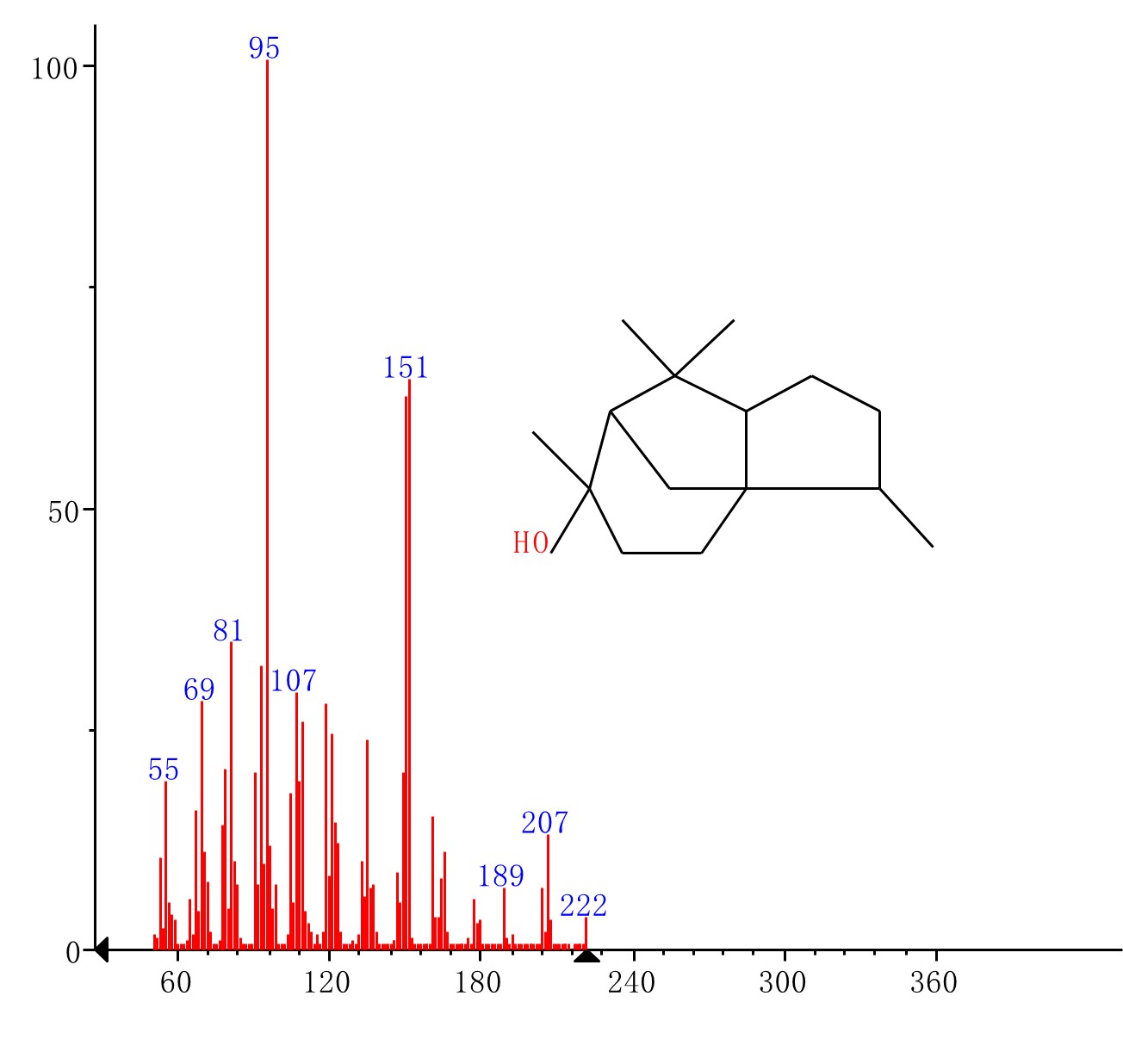

Supplement: Supplemental Information 7 [file peerj-11-15818-s007.zip › GC/TPS9-21A FPP/A7.jpg]

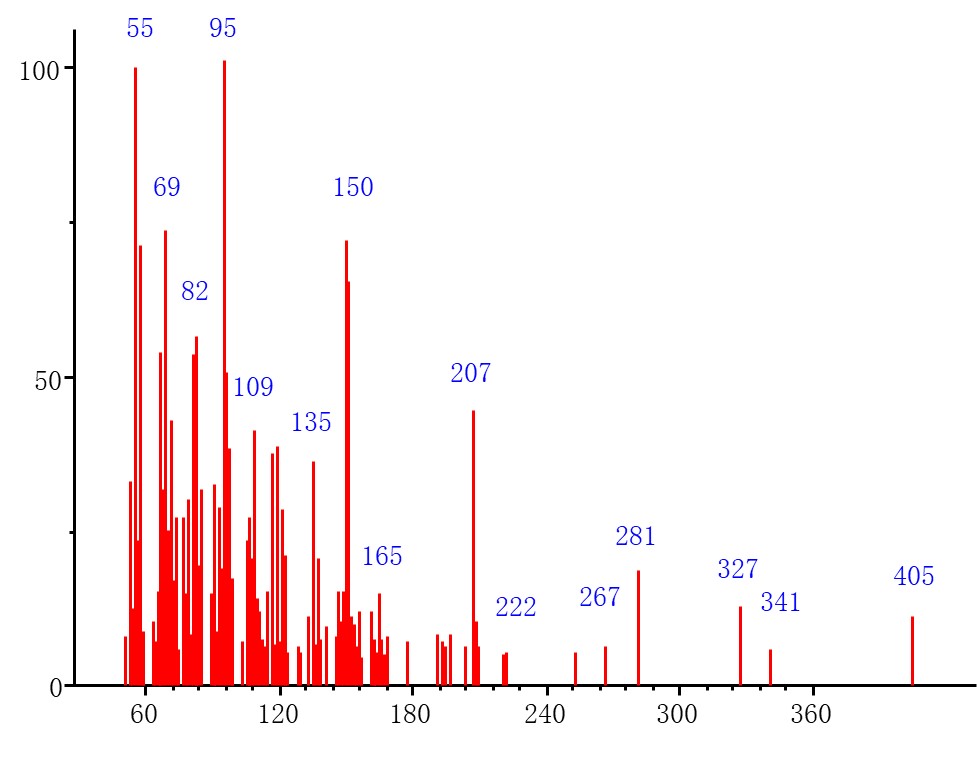

Supplement: Supplemental Information 7 [file peerj-11-15818-s007.zip › GC/TPS9-21A FPP/A8.jpg]

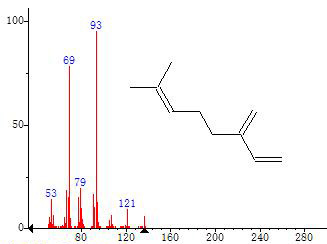

Supplement: Supplemental Information 7 [file peerj-11-15818-s007.zip › GC/TPS9-21a GPP/A1.jpg]

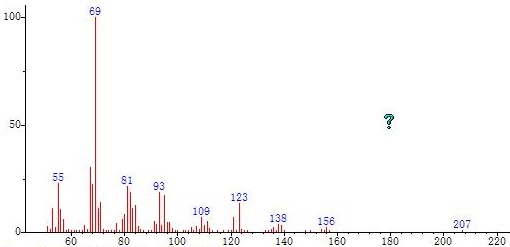

Supplement: Supplemental Information 7 [file peerj-11-15818-s007.zip › GC/TPS9-21a GPP/A10.jpg]

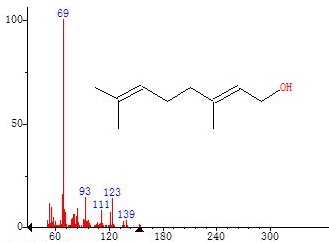

Supplement: Supplemental Information 7 [file peerj-11-15818-s007.zip › GC/TPS9-21a GPP/A11.jpg]

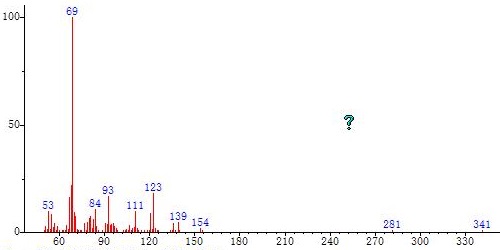

Supplement: Supplemental Information 7 [file peerj-11-15818-s007.zip › GC/TPS9-21a GPP/A12.jpg]

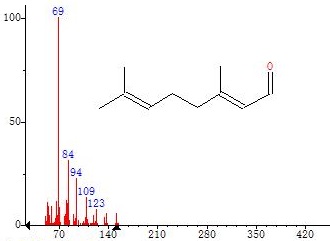

Supplement: Supplemental Information 7 [file peerj-11-15818-s007.zip › GC/TPS9-21a GPP/A13.jpg]

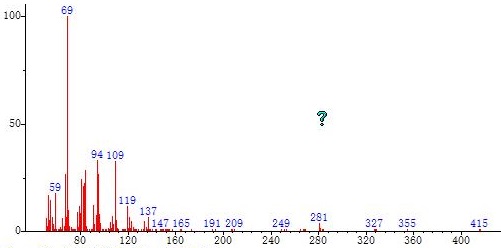

Supplement: Supplemental Information 7 [file peerj-11-15818-s007.zip › GC/TPS9-21a GPP/A14.jpg]

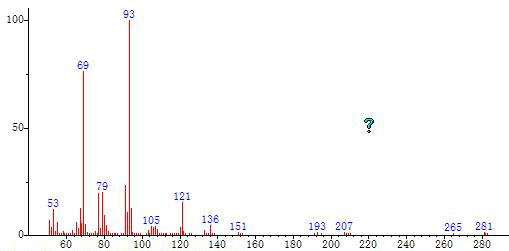

Supplement: Supplemental Information 7 [file peerj-11-15818-s007.zip › GC/TPS9-21a GPP/A2.jpg]

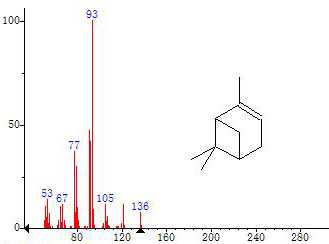

Supplement: Supplemental Information 7 [file peerj-11-15818-s007.zip › GC/TPS9-21a GPP/A3.jpg]

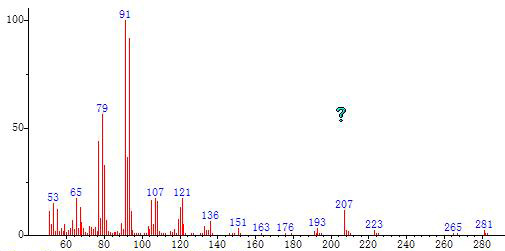

Supplement: Supplemental Information 7 [file peerj-11-15818-s007.zip › GC/TPS9-21a GPP/A4.jpg]

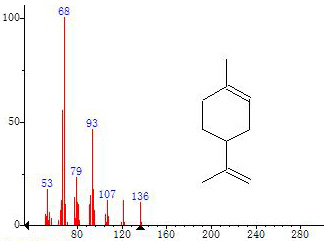

Supplement: Supplemental Information 7 [file peerj-11-15818-s007.zip › GC/TPS9-21a GPP/A5.jpg]

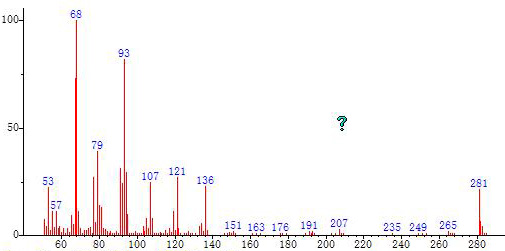

Supplement: Supplemental Information 7 [file peerj-11-15818-s007.zip › GC/TPS9-21a GPP/A6.jpg]

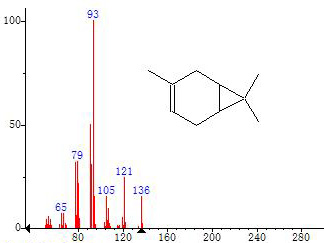

Supplement: Supplemental Information 7 [file peerj-11-15818-s007.zip › GC/TPS9-21a GPP/A7.jpg]

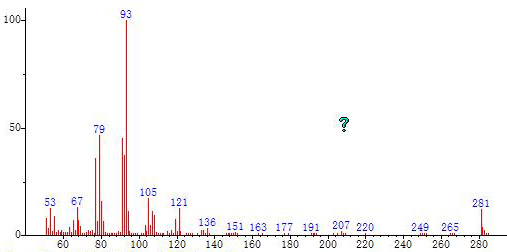

Supplement: Supplemental Information 7 [file peerj-11-15818-s007.zip › GC/TPS9-21a GPP/A8.jpg]

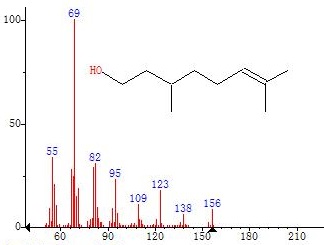

Supplement: Supplemental Information 7 [file peerj-11-15818-s007.zip › GC/TPS9-21a GPP/A9.jpg]
