# Supplementary material for: Identification and characterization of novel sesquiterpene synthases TPS9 and TPS12 from Aquilaria sinensis
Source: PeerJ. 2023 Aug 30;11:e15818. doi: 10.7717/peerj.15818 (PMC10474832; doi:10.7717/peerj.15818)
Supplement: Supplemental Information 3 [file peerj-11-15818-s003.docx]

**Table 1:**

**Summary information of physiological and biochemical properties of the TPS9 and TPS12 proteins.**

| Name | Gene length | Animo  Acids | MW (KDa) | PI | GRVAY | Instability Index | Aliphatic  Index | Subcellular localization | | |
| --- | --- | --- | --- | --- | --- | --- | --- | --- | --- | --- |
|  |  |  |  |  |  |  |  | Wolf Psort | Predotar | SCLpredT |
| TPS9 | 1383 | 460 | 53.3 | 5.78 | -0.217 | 42.58 | 98.20 | cyto: 7,  nucl: 6  chlo: 1 | mitochondrial: 1%, plastid: 1%, elsewhere: 99% | cytoplasm |
| TPS12 | 1632 | 543 | 62.9 | 5.24 | -0.271 | 48.78 | 100.26 | cyto: 11, chlo: 1, nucl: 1, pero: 1 | Mitochondrial:1%, elsewhere: 99% | cytoplasm |
